# Nonlinear association of BMI with all-cause and cardiovascular mortality in type 2 diabetes mellitus: a systematic review and meta-analysis of 414,587 participants in prospective studies

**DOI:** 10.1007/s00125-016-4162-6

**Published:** 2016-11-25

**Authors:** Francesco Zaccardi, Nafeesa N. Dhalwani, Dimitris Papamargaritis, David R. Webb, Gavin J. Murphy, Melanie J. Davies, Kamlesh Khunti

**Affiliations:** 1 Diabetes Research Centre, Leicester Diabetes Centre, University of Leicester, Leicester General Hospital, Leicester, LE5 4PW UK; 2 University of Leicester, Department of Cardiovascular Sciences, Clinical Sciences Wing, Glenfield General Hospital, Leicester, LE3 9QP UK

**Keywords:** BMI, Body mass index, Cardiovascular disease, Diabetes, Meta-analysis, Mortality, Nonlinearity, Systematic review

## Abstract

**Aims/hypothesis:**

The relationship between BMI and mortality has been extensively investigated in the general population; however, it is less clear in people with type 2 diabetes. We aimed to assess the association of BMI with all-cause and cardiovascular mortality in individuals with type 2 diabetes mellitus.

**Methods:**

We searched electronic databases up to 1 March 2016 for prospective studies reporting associations for three or more BMI groups with all-cause and cardiovascular mortality in individuals with type 2 diabetes mellitus. Study-specific associations between BMI and the most-adjusted RR were estimated using restricted cubic splines and a generalised least squares method before pooling study estimates with a multivariate random-effects meta-analysis.

**Results:**

We included 21 studies including 24 cohorts, 414,587 participants, 61,889 all-cause and 4470 cardiovascular incident deaths; follow-up ranged from 2.7 to 15.9 years. There was a strong nonlinear relationship between BMI and all-cause mortality in both men and women, with the lowest estimated risk from 31–35 kg/m^2^ and 28–31 kg/m^2^ (*p* value for nonlinearity <0.001) respectively. The risk of mortality at higher BMI values increased significantly only in women, whilst lower values were associated with higher mortality in both sexes. Limited data for cardiovascular mortality were available, with a possible inverse linear association with BMI (higher risk for BMI <27 kg/m^2^).

**Conclusions/interpretation:**

In type 2 diabetes, BMI is nonlinearly associated with all-cause mortality with lowest risk in the overweight group in both men and women. Further research is needed to clarify the relationship with cardiovascular mortality and assess causality and sex differences.

**Electronic supplementary material:**

The online version of this article (doi:10.1007/s00125-016-4162-6) contains peer-reviewed but unedited supplementary material, which is available to authorised users.

## Introduction

Compared with the general population, diabetes mellitus is associated with a higher mortality, mainly attributable to cardiovascular causes [1]. In 2012, an estimated 1.5 million deaths were directly caused by diabetes and by 2030 diabetes is expected to be the seventh leading cause of death worldwide [2]. Being overweight or obese is one of the main modifiable risk factors for type 2 diabetes mellitus, and obesity has been significantly associated with an increased mortality risk in the general population [3]. However, evidence on the association between obesity and mortality in patients with diabetes remains inconclusive with some studies reporting an inverse association between obesity (estimated using the conventional measure of BMI) and mortality [4–6], some reporting U-shaped associations [7, 8], some reporting linear positive associations [9, 10], and some reporting no association [11].

A recent study investigating the relationship between BMI and mortality in 10,568 people with type 2 diabetes found a lower mortality risk in overweight (BMI ≥25 kg/m^2^), higher mortality risk in underweight (≤18.5 kg/m^2^), and a similar mortality risk in obese (BMI ≥30 kg/m^2^) people compared with those with normal weight, indicating a nonlinear association between BMI and all-cause mortality [12]. A recent systematic review including nine studies reported a reduced risk of all-cause mortality in overweight and obese people with type 2 diabetes when compared with normal or non-overweight people and a 5% progressive decrease in mortality for every 5 kg/m^2^ increase in BMI [13]. This analysis, however, did not explore whether a nonlinear relationship exists between BMI and outcomes. This is particularly relevant, given the possible presence of nonlinearity for BMI values within the category of overweight or obesity. Moreover, a clearer determination of the relationship across BMI values would elucidate the comparative relevance of higher and lower BMI values on mortality risk in people with diabetes.

To help clarify the evidence, we conducted a systematic review and meta-analysis of prospective studies to examine the shape of association of BMI with all-cause and cardiovascular mortality in individuals with type 2 diabetes mellitus.

## Methods

This meta-analysis was performed following the Meta-analysis Of Observational Studies in Epidemiology (MOOSE) recommendations [14].

### Data sources, searches and study selection

Three independent investigators searched for prospective studies reporting associations between BMI and all-cause and cardiovascular mortality in people with type 2 diabetes mellitus using the databases PubMed, Web of Science, and Scopus. The search strategy combined keywords related to the exposure (i.e. ‘obesity’ OR ‘body mass index’ OR ‘BMI’), population (i.e. ‘diabetes’ OR ‘type 2 diabetes’), outcome (i.e. ‘cardiovascular’ OR ‘vascular’ OR ‘mortality’) and study design (i.e. ‘cohort’ OR ‘longitudinal’) and included articles published in English before 1 March 2016 (details of the search strategy are reported in the electronic supplementary material [ESM]). Reference lists of retrieved articles were also manually scanned for all relevant additional studies and reviews. Prospective studies were included if the RR of cardiovascular or all-cause mortality was reported for at least three BMI categories (one referent and two nonreferent). When multiple publications reported associations from the same cohort, we included the one with the longest follow-up or the largest sample size. We excluded studies including only participants with type 1 diabetes, or only participants with prevalent cardiovascular disease at baseline, or hospitalised patients. If studies reported estimates stratified by prevalent cardiovascular disease at baseline, we used data for participants without baseline cardiovascular disease.

### Data extraction and quality assessment

We used standardised, pre-defined forms for data extraction and quality assessment. We abstracted data on first author name; year of journal publication; study location and follow-up duration; population age, source, sex distribution; baseline prevalence of cardiovascular disease; exposure definition and assessment; endpoint definition and ascertainment; and adjustment level. For each reported category of BMI, we collected data on: mean or median BMI value; number of participants (or person-years of follow-up); number of cases; and the RR with 95% CI. If category-specific mean or median BMI was not reported, we assumed the BMI value to be the midpoint between lower and upper boundaries; if one boundary was not reported in the first or last category, we assumed the difference between boundaries to be equal to that of the adjacent category. When studies published more than one adjusted RR, we extracted the most-adjusted estimate. When reported, we extracted data separately for men and women. We contacted study authors when it was not possible to extract data from published reports. Study quality was assessed by two authors using the nine-star Newcastle–Ottawa Scale (NOS) [15] and discrepancies were resolved by consensus or independent arbitration.

## Data synthesis and analysis

For each outcome, we extracted RRs with 95% CIs for men, women and both. When studies only reported association separately for men and women, a within-study summary estimate was computed by fixed-effect meta-analysis. A similar summary estimate was calculated for one study reporting associations stratified by age. To assess the relationship between BMI and outcomes, we performed a two-stage random-effects dose–response meta-analysis [16–18]. In the first stage, we modelled BMI values using restricted cubic splines with three knots at 10%, 50% and 90% percentile of the BMI distribution. We then estimated study-specific trend between RRs and the two BMI spline transformations with a generalised least squares method, which accounts for the correlation within each set of RRs [16]. In the second stage, the two study-specific regression coefficients were combined in a multivariate random-effects meta-analysis using the restricted maximum likelihood method [19]. We used coefficients obtained from multivariate meta-analysis to perform a Wald-type test for the hypothesis of no exposure–disease association (both regression coefficients equal to zero) and of nonlinearity (second spline coefficient equal to zero) [20]. We assessed heterogeneity between sexes with a multivariate meta-regression. Publication bias was estimated with the Egger’s test [21]. We conducted a sensitivity analysis excluding studies that only report unadjusted effect estimates to assess whether the associations between BMI and mortality change. Furthermore, the association between BMI and several chronic conditions has been reported to vary in different demographic regions; therefore we also assessed whether there was any statistically significant heterogeneity in the association between BMI and mortality by regions (USA, Europe and UK, Asia) using multivariate meta-regression and also stratified the analysis by regions. In addition, use of medications may affect the mortality risk, and considering the changes in the guidelines for the management of diabetes and use of glucose-lowering therapies over the study period we conducted a multivariate meta-regression categorising the study periods as 2003–2010, 2011–2013, 2014–2015) to assess whether there were significant differences in the effect estimates by the time of the study. We also performed additional sensitivity analyses with three knots at different positions (e.g. 25th, 50th and 75th percentile) to assess whether the shape of the association changed.

Analyses were performed using Stata 14.1 [22, 23] and R [24, 25]. Two-sided *p* value <0.05 was considered statistically significant.

## Results

### Study characteristics

**Fig. 1:**
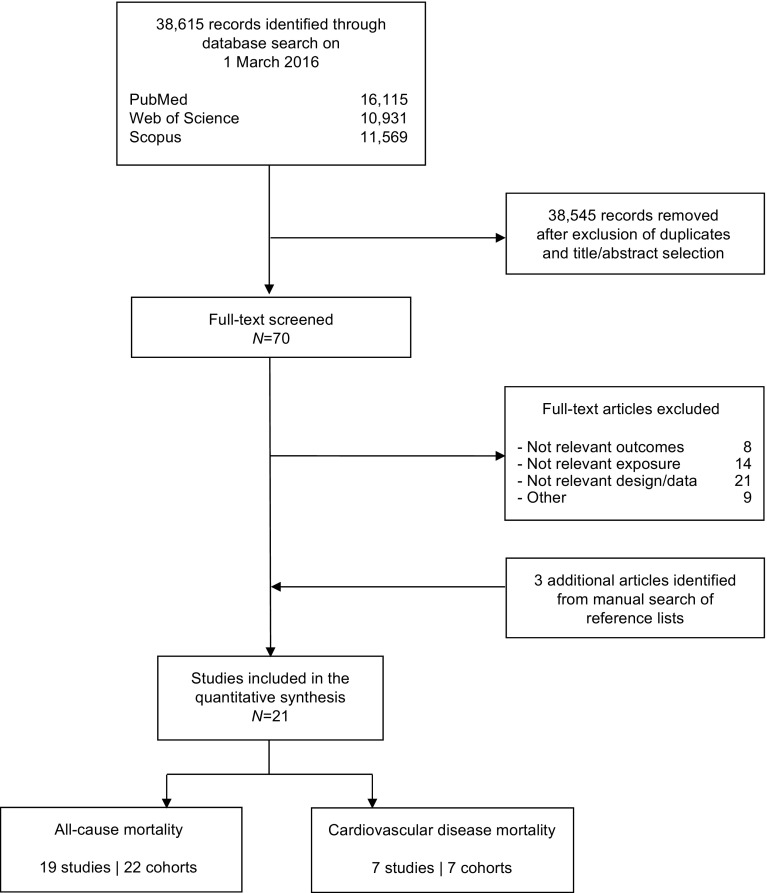
Study flow diagram in line with Preferred Reporting Items for Systematic Reviews and Meta-Analyses (PRISMA) recommendations

Our initial search yielded 38,615 articles. After screening of title/abstract and exclusion of duplicates, 70 articles remained for further evaluation (Fig. 1). Following detailed assessments, and after the inclusion of three articles identified from manual searches, 21 articles were included in the quantitative analysis (21 studies and 24 unique cohorts; Table 1) [7, 8, 11, 15, 26–42]. Study characteristics and the NOS scores are provided in ESM Tables 1–3. Out of the 21 studies included, ten (48%) had an NOS score of 8, whilst two (10%) studies had NOS scores of 9 or 5, indicating that most of the studies included were of good or high quality. Overall, information was available for 414,587 male and female participants with 61,889 and 4470 incident all-cause and cardiovascular mortality events, respectively. In nine studies associations were reported separately for men and women (Table 1). The mean (or median) duration of follow-up ranged from 2.7 to 15.9 years and the mean baseline age ranged from 40 to 77 years. All but three studies adjusted for age and sex (when appropriate) and diagnosis of diabetes was mainly self-reported or based on biochemical measurements (ESM Tables 1 and 2). Ten studies (47.6%) with 214,694 participants (51.8%) included people from the USA or UK, whilst seven studies were from elsewhere in Europe, one multinational, and one each from Iran, South Korea, and Taiwan.

**Table 1:** Characteristics of the included studies

						Number of events		
Study First author [Ref]	Country, year	Male (%)	Mean age (years)^a^	Mean follow-up (years)	Number of participants	All-cause mortality	CVD mortality	Stratified by sex
Bozorgmanesh [26]	Iran, 2014	44.6	53.6	9.1^b^	1322	108	–	–
Chaturvedi [27]^c^	Multinational, 1995	47.3	47.2	12.0	2740	544	–	•
Church [28]	US, 2005	100	50.0	15.9	2316	–	179	–
Costanzo [15]	UK, 2015	54.0	63.0^b^	10.6^b^	10,568	3744	–	–
Eeg-Olofsson [29]	Sweden, 2009	55.7	60.3	5.6	13,087	664	–	–
Jackson [30]^d^	US, 2014	40.0	57.2	9.0^e^	2035	247	–	–
Khalangot [11]	Ukraine, 2009	33.6	64.7	2.7	81,603	6570	2677	•
Kokkinos [7]	US, 2012	100	60.0	7.5^b^	4156	1074	–	–
Logue [31]^f^	Scotland, 2013	54.7	59.5	4.7	106,640	9631	–	•
Ma [32]^f^	South Korea, 2012	39.2	57.1	9.2	845	–	50	•
McEwen [8]^f^	US, 2007	47.0	61.0	3.7	8445	758	322	•
Menke [33]	US, 2014	50.7	56.9	6.5	2543	668	259	–
Murphy [34]	Iceland, 2014	55.3	77.4	6.7^b^	637	188	–	–
Perotto [35]^f^	Italy, 2013	43.8	68.7	10.2^b^	1475	972	498	–
Sluik [36]	Europe, 2011	53.8	57.3	9.3^b^	5435	641	–	•
Thomas [37]	UK, 2014	53.0	60.0	5.0^b^	37,272	1762	–	–
Tobias [38]	US, 2014	21.5	61.4	15.8	11,427	3083	–	•
Tseng [39]	Taiwan, 2012	46.0	60.7	12.0	89,056	26,951	–	•
Tuomilehto [40]	Malta, 1994	40.0	>40	5.0	295	39	–	•
Zhao [41]^f,g^	US, 2014	37.8	52.3	8.7	29,292	3033	–	–
Zoppini [42]	Italy, 2003	48.2	65.3	10.0	3398	1212	485	–

^a^When not reported for the overall population, the value has been estimated as weighted mean

^b^Median

^c^Three cohorts

^d^Non-smokers

^e^Maximum follow-up

^f^Additional data available from correspondence

^g^Two cohorts

### All-cause mortality

**Fig. 2:**
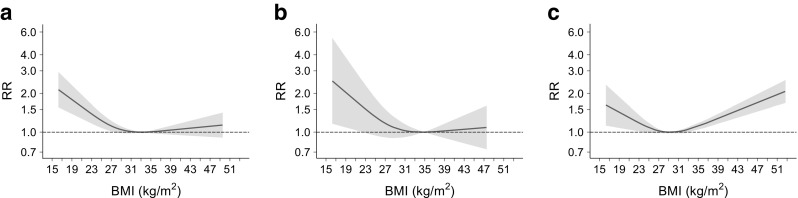
Relationship between BMI and all-cause mortality for (**a**) both sexes, (**b**) men and (**c**) women. Grey shading indicates 95% CI

Overall, 18 studies comprising 407,270 participants and 60,815 all-cause death events were included in the analysis; study-specific associations are reported in ESM Figs 1–3. As shown in Fig. 2, there was an overall nonlinear relationship between BMI and all-cause mortality (*p* value for no exposure–disease association <0.001; *p* value for nonlinearity <0.001). The lowest risk was at BMI around 33 kg/m^2^, with an increased mortality risk more evident for lower than higher BMI values. Although no statistically significant heterogeneity was found (*p* = 0.376), the shape of the relationships differed between men and women. In the analysis restricted to men (nine studies, 141,709 participants/24,230 events), the lowest risk was between 31 and 35 kg/m^2^, with an increased risk for values lower than 31 kg/m^2^ and a slightly non-significant increase for values higher than 35 kg/m^2^ (*p* value for no exposure–disease association 0.0034; *p* value for nonlinearity 0.001). Conversely, almost a symmetrical increase was evident for women (eight studies, 168,088 participants/25,061 events) with a BMI nadir around 28–31 kg/m^2^ (*p* value for no exposure–disease association <0.001; *p* value for nonlinearity <0.001).

For the three analyses, there was evidence of heterogeneity across studies (ESM Table 4) but not of publication bias (*p* values 0.403; 0.521; and 0.550, respectively).

### Cardiovascular mortality

**Fig. 3:**
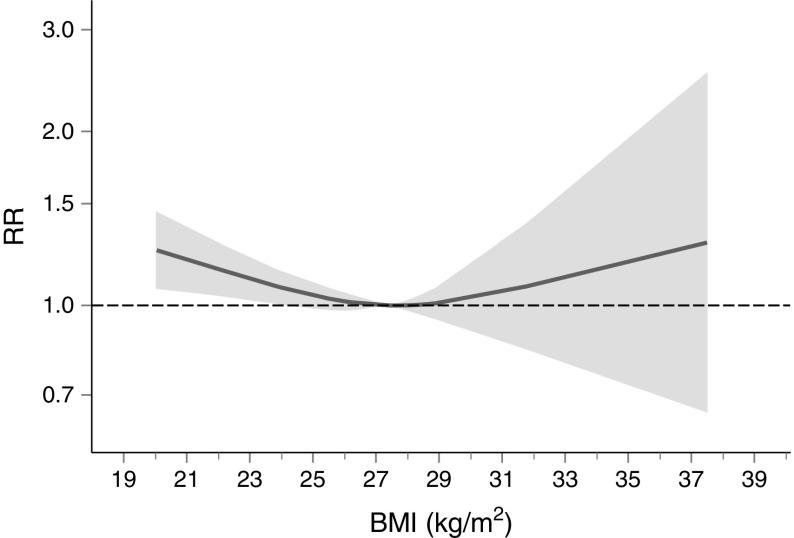
Relationship between BMI and cardiovascular mortality (both sexes). Grey shading indicates 95% CI

Six studies comprising 98,309 participants reported data on BMI and 4291 cardiovascular incident deaths for both men and women (Table 1 and ESM Fig. 4). The overall shape of the association suggested an increased risk of cardiovascular mortality for values lower than 27 kg/m^2^ and a less clear association for higher values (*p* value for no exposure–disease association 0.0176; *p* value for nonlinearity 0.140) (Fig. 3). However, a reduced risk for values greater than 27 kg/m^2^ and a possible linear negative trend cannot be excluded (RR per unit increase of BMI: 0.98; 95%CI: 0.96, 0.99; *p* = 0.013). No publication bias was present (*p* = 0.258). Owing to the limited number of studies, it was not possible to stratify the analysis by sex.

### Sensitivity analyses

The results were consistent with those of the main analysis after the exclusion of two studies reporting unadjusted estimates, yet the risk of all-cause death for high values of BMI in women was less pronounced (ESM Table 5 and ESM Fig. 5). No heterogeneity was found in stratified analyses by region (USA, Europe and UK, Asia; *p* = 0.069) or period (2003–2009, 2011–2013, 2014–2015; *p* = 0.117) (ESM Table 6 and ESM Fig. 6). Results were substantially similar in sensitivity analyses considering alternative knot locations (ESM Fig. 7 and ESM Fig. 8).

## Discussion

Results of this meta-analysis indicate a clear nonlinear relationship between BMI and all-cause mortality, with the lowest risk found around 33 kg/m^2^ and an increased risk more evident for lower compared with higher values. The shape of the association between BMI and all-cause mortality, however, was found to be different between male and female participants such that the risk did not increase for values higher than 35 kg/m^2^ in men whilst a symmetrical rise was found in women above and below a nadir around 28–31 kg/m^2^. Conversely, the limited data availability for cardiovascular mortality precluded a clear interpretation of the relationship, particularly for high values of BMI.

Our meta-analysis is the largest to-date to assess the association between BMI and mortality among people with diabetes. However, there are certain limitations of this study. First, most of the included studies adjusted risk estimates for a number of potential confounders; only five, however, adjusted for alcohol consumption, which is closely associated with mortality and BMI [43, 44]. Cardiorespiratory fitness has been reported as a potential effect modifier in the association of BMI and mortality, suggesting a ‘fit and fat’ phenomenon whereby higher cardiorespiratory fitness attenuates the increased mortality risk associated with higher BMI [45]. Given the available data, we could not explore this hypothesis in our meta-analysis. Similarly, we could not assess the impact of fat distribution, another possible effect modifier in the relationship between BMI and outcomes. Indeed, recent research has suggested that fat distribution is more strongly associated than BMI to future cardiovascular events risk [46]. Possible confounders of the association are drug treatment (as some glucose-lowering drugs are associated with both body weight and mortality [47]) and smoking, which is negatively correlated with BMI and positively with mortality [48, 49]. This association is conditioned on the effect of having diabetes such that the inverse association between smoking and BMI is exaggerated, which may potentially lead to an underestimation of the mortality risk associated with obesity. This may result in a collider bias, requiring detailed adjustment for smoking [50]. Almost all studies included in the meta-analysis, however, adjusted for smoking status. We were unable to conduct a stratified analysis by smoking status as only two studies reported the risk of mortality by BMI separately for each smoking category. Zhao and colleagues found that the risk estimates for different BMI groups compared with a reference group (30–34.9 kg/m^2^) were slightly higher in never smokers in comparison with current smokers in both Black and White participants [41]. In comparison Jackson and colleagues did not find a variation in the association between BMI and mortality by smoking status [30].

Our findings showed a nonlinear relationship between BMI and all-cause mortality in people with diabetes, with the lowest risk around 33 kg/m^2^. This is in line with a large meta-analysis of 141 studies assessing the association between BMI and mortality in the general population which found a 6% statistically significant reduction in mortality risk associated with being overweight (BMI 25–30 kg/m^2^), a 5% non-significant reduction associated with being modestly obese (BMI 30–35 kg/m^2^), and a 29% increase in the risk associated with a BMI of over 35 kg/m^2^ when compared with normal weight (BMI 18–25 kg/m^2^) [3]. In contrast, a recent dose–response meta-analysis assessing the relationship in type 2 diabetes reported a progressive linear 5% reduction of all-cause mortality for every 5 kg/m^2^ increase in the BMI (six studies and eight cohorts) [13]. However, this meta-analysis also included studies reporting on populations with pre-existing cardiovascular morbidities, such as heart failure [51, 52], whilst some large population-based studies were omitted. Moreover, in view of previous knowledge about the shape of the association between BMI and mortality in the general population, we did not assume the dose–response relationship to be linear and did not use conventional BMI categories but rather investigated possible nonlinearity in the BMI–mortality association. This enabled us to clarify the shape of the association across a wide range of BMI and assess whether there was a significant departure from linearity and a difference between male and female participants. Our results, indeed, clearly showed a nonlinear relationship for all-cause mortality, whilst the association with cardiovascular mortality is less clear (and potentially linear). We found, in particular, the mortality curve to be steeper in women compared with men, with a considerably higher risk of mortality at higher BMIs in women than men, and the nadir to be lower in women than men. Of note, in the sensitivity analysis excluding studies reporting unadjusted estimates, the risk for higher BMI was less pronounced: whether this is relates to a reduced statistical power or a true effect could not be ascertained. However, our findings are line with a general population study including over 12 million adults from South Korea, which found the optimal BMI for women to be lower than men, especially at younger ages [53]. This may be attributed to a positive and strong association between obesity and sex-specific cancer incidence and mortality in women [54–57].

The progressively increasing risk of all-cause mortality for values of BMI ≤25 kg/m^2^ in both men and women may have a number of explanations. Sarcopenic obesity is a condition characterised by relative increase in fat mass and reduction in muscle mass, thus resulting in a different total body fat composition for the same weight (and therefore BMI). Typically, people with sarcopenic obesity have visceral fat accumulation [58], a well-known risk factor for cardiovascular mortality [46]. The prevalence of sarcopenic obesity has been reported to be higher in type 2 diabetes compared with non-diabetes (15.7% vs 6.9% respectively in the Korean Sarcopenic Obesity Study) [59] and it has also been linked to an increased risk of falls [59], physical disability [60] and cardiovascular and all-cause mortality [61–64]. Another possible explanation could be reverse causation. Underlying conditions may result in loss of appetite or increased metabolic demands with subsequent unintentional weight loss. This phenomenon has been reported to be stronger in people with diabetes as they have higher rates of underlying illness compared with the general population [50]. This may partly explain the shape of association in our meta-analysis, with an increased risk for low values of BMI. In this analysis we were able to include data from eight studies that excluded the initial 2 years of follow-up to reduce the risk of reverse causation. A third possible reason is genetics. Some single nucleotide polymorphism (SNP) variants are more strongly associated with type 2 diabetes in lean compared with obese subsets [65]. Among such SNPs, variants at TCF7L2 and CDKAL1 have also been associated with increased risk of cancer in people with diabetes [66], potentially explaining the higher mortality risk for low BMI values. However, stronger evidence (ideally with Mendelian randomisation studies) is needed to support a genetic explanation in people with diabetes. Finally, potentially improved diagnosis and care of obese people with type 2 diabetes (i.e. screen-detected diabetes, more intensive control of risk factors) is another plausible explanation [67].

### Conclusion

Findings from this meta-analysis of prospective cohort studies demonstrated a nonlinear relationship between BMI and all-cause mortality, with lower risks between 31 and 35 kg/m^2^ for men and 28 and 31 kg/m^2^ for women. Further research is needed to decipher whether this association is truly causal and different from that between BMI and cardiovascular mortality. Furthermore, to better clarify the link between excess fat and outcomes in people with diabetes, multiple assessments (including overall and region-specific body fat accumulation) over time are warranted. The results of our study, along with available previous knowledge in this field, do not downgrade the importance of weight control and appropriate lifestyle as cornerstones for the prevention and management of cardiometabolic diseases.

## Electronic supplementary material

Below is the link to the electronic supplementary material.

ESM (PDF 1220 kb)

## Abbreviations

NOS: Newcastle–Ottawa Scale
SNP: Single nucleotide polymorphism
