# Supplementary material for: Nonlinear association of BMI with all-cause and cardiovascular mortality in type 2 diabetes mellitus: a systematic review and meta-analysis of 414,587 participants in prospective studies
Source: Diabetologia. 2016 Nov 25;60(2):240–8. doi: 10.1007/s00125-016-4162-6 (PMC6518080; doi:10.1007/s00125-016-4162-6)
Supplement: Supplementary file 1 — (PDF 1220 kb) [file 125_2016_4162_MOESM1_ESM.pdf]

ELECTRONIC SUPPLEMENTARY MATERIAL

Nonlinear association of BMI with all-cause and cardiovascular mortality in type 2 diabetes mellitus: a systematic review and meta-analysis of 414,587 participants in prospective studies

Francesco Zaccardi[1], Nafeesa N Dhalwani[1], Dimitris Papamargaritis[1], David Webb[1], Gavin J Murphy[2], Melanie J Davies[1], Kamlesh Khunti[1]

[1] University of Leicester, Diabetes Research Centre, Leicester Diabetes Centre, Leicester General Hospital, Leicester LE5 4PW
[2] University of Leicester, Department of Cardiovascular Sciences, Clinical Sciences Wing, Glenfield General Hospital, Leicester LE3 9QP

Search Strategy

Supplementary Tables: 6

Supplementary Figures: 8

Search Strategy

PubMed, Web of Science, and Scopus articles published in English before March 1st, 2016 were identified. Electronic search was performed independently by three Authors and supplemented by scanning reference lists of all relevant articles, including reviews, by hand searching of relevant journals. We used terms related to exposure, population, outcomes, and study design.

Exposure: Obesity OR body mass index OR bmi OR overweight OR body weight OR paradox
Population: Diabetes OR type 2 diabetes OR NIDDM NOT type 1 diabetes NOT IDDM NOT pregnancy
Outcome: Cardiovascular OR vascular OR mortality OR stroke OR cerebrovascular disease OR death OR mortality
Study design: Cohort OR prospective OR longitudinal OR hazard OR risk OR odds
Limits: Humans

For PubMed the search was

(Obesity[Title/Abstract] OR body mass index[Title/Abstract] OR bmi[Title/Abstract] OR overweight[Title/Abstract] OR body weight[Title/Abstract] OR paradox[Title/Abstract])

AND

(Diabetes[Title/Abstract] OR type 2 diabetes[Title/Abstract] OR NIDDM[Title/Abstract])

NOT

(Type 1 diabetes[Title/Abstract] OR IDDM[Title/Abstract] OR pregnancy[Title/Abstract])

AND

(Cardiovascular[Title/Abstract] OR vascular[Title/Abstract] OR stroke[Title/Abstract] OR cerebrovascular disease[Title/Abstract] OR death[Title/Abstract] OR mortality[Title/Abstract])

AND

(Cohort[All Fields] OR ("longitudinal studies"[MeSH Terms] OR ("longitudinal"[All Fields] AND "studies"[All Fields]) OR "longitudinal studies"[All Fields] OR "prospective"[All Fields]) OR longitudinal[All Fields] OR hazard[All Fields] OR ("risk"[MeSH Terms] OR "risk"[All Fields]) OR odds[All Fields])

AND

"humans"[MeSH Terms]

Search strategy was specifically translated for each database.

ESM Table S1: Summary of the studies included in the meta-analysis

Study<br>First Author	Population<br>Sample - *Country*	Exposure<br>definition	Exposure<br>assessment	Endpoint<br>definition	Endpoint<br>ascertainment	Confounders
Bozorgmanesh	Population from Tehran lipid and glucose study<br>*IRAN*	BMI thirds<br><br>Median BMI by thirds:<br>1st: 24·9 kg/m^2<br>2nd: 28·9 kg/m^2<br>3rd: 33·8 kg/m^2	Weight was measured with subjects minimally clothed without shoes using digital scale and recorded to nearest 0·1 kg. Height was measured in standing position without shoes, using tape measure while shoulders in normal alignment	All-cause mortality	Preliminary data collected by phone. Complimentary data collected from hospital or death certificate	Sex, age, systolic blood pressure, blood pressure lowering medications, total and HDL cholesterol, smoking, history of previous CVD, lifestyle modification intervention, waist circumference
Chaturvedi	Centres in Europe, East Asia and USA. Random sampling stratified from a list of all clinic attenders in a 1-year period<br>*MULTINATIONAL*	BMI thirds<br><br>Europeans<br><26 kg/m^2<br>26 to <29 kg/m^2<br>≥29 kg/m^2<br><br>Native Americans<br><29 kg/m^2<br>29 to <34 kg/m^2<br>≥34 kg/m^2<br><br>East Asians<br><22 kg/m^2<br>22 to <25 kg/m^2<br>≥25 kg/m^2	Height and weight were recorded at the clinic without shoes and outdoor clothing	All-cause mortality	Information from death certificates, hospital records and post-mortem records.	Systolic blood pressure, age, duration of diabetes, serum cholesterol, smoking status, retinopathy, insulin therapy
Church	Population from the Aerobics Center Longitudinal Study. T1DM and T2DM patients at baseline without a history of stroke or myocardial infarction who had a complete baseline evaluation at outpatient clinic between 1970 and 1988<br>*USA*	BMI categories<br><br>18·5–24·9 kg/m^2<br>25·0–29·9 kg/m^2<br>30–34·9 kg/m^2	BMI was computed from weight and height measured in the clinic	CVD mortality	Deaths identified from the National Death Index (NDI). The underlying cause of death was determined from the NDI report or by a nosologist's review of official death certificates. CVD mortality was defined according to ICD-9 codes	Unadjusted (Odds Ratios estimated)
Costanzo	Patients who attend the Outpatient clinic in Kingston Upon Hull with T2DM were enrolled in a registry between 1995-2005<br>*UK*	BMI categories<br><br><18·5 kg/m^2<br>18·5–24·9 kg/m^2<br>25–29·9 kg/m^2<br>30–34·9 kg/m^2<br>≥35 kg/m^2	Data on weight and height collected at the initial visit by medical and nursing staff	All-cause mortality	National register	Age, sex, duration of T2DM, smoking history, systolic blood pressure, COPD, cancer, CKD, previous CVD.
Eeg-Olofsson	Patients sampled from the Swedish National Diabetes Register between 1998 and 2003<br>*SWEDEN*	BMI categories<br><br><25 kg/m^2<br>25–29·9 kg/m^2<br>≥30 kg/m^2	Clinical characteristics including BMI collected at baseline	All-cause mortality	Deaths retrieved through linkage with the Swedish Cause of Death and Hospital Discharge Registers	Age, sex, smoking ,type of hypoglycaemic medications, diabetes duration, systolic blood pressure, antihypertensive drug, lipid-lowering drugs, microalbuminuria, HbA1c
Jackson	Population data from the National Health Interview Survey (never smokers, with no cancer or heart disease)<br>*USA*	BMI categories<br><br>15·02–22·83 kg/m^2<br>22·84–25·09 kg/m^2<br>25·1–27·46 kg/m^2<br>27·47–31·02 kg/m^2<br>31·03–54·92 kg/m^2	Self-reported weight and height extracted from the Survey	All-cause mortality	Deaths identified by linking to the National Death Index	Age, race, education, marital status, alcohol consumption, leisure time physical activity, general health status, income, region of country, self-reported diabetes medication and general health status, diabetes duration

ESM Table S1 (cont'd): Summary of the studies included in the meta-analysis

Study First Author	Population Sample	Exposure definition	Exposure assessment	Endpoint definition	Endpoint ascertainment	Confounders
Khalangot	Patients receiving insulin or oral glucose lowering drug extracted from System of Diabetes Mellitus Care in Ukraine (No history of CHD or stroke)<br>*UKRAINE*	BMI categories<br>$<23·0$ kg/m^2<br>23·0–24·9 kg/m^2<br>25·0–29·9 kg/m^2<br>30·0–34·9 kg/m^2<br>$\geq35·0$ kg/m^2	Data on weight and height collected at the initial visit by medical staff	All-cause mortality<br>CVD mortality	Data linked through computerised registers. Mortality data obtained from primary care doctors with causes of death	Age, smoking, alcohol consumption, systolic blood pressure, total cholesterol, diabetes treatments, duration of diabetes
Kokkinos	Patients with T2DM who performed symptom-limited exercise tolerance test (Veterans Affairs Medical Centers)<br>*USA*	BMI categories<br>18·5–24·9 kg/m^2<br>25·0–29·9 kg/m^2<br>30–34·9 kg/m^2<br>≥35 kg/m^2	BMI was obtained from subjects' computerized medical records. Standardised scale used to assess body weight and height	All-cause mortality	Data and dates of death verified from the Veterans Affairs Beneficiary Identification and Record Locator System File	Age, history of CVD, race, hypertension, dyslipidaemia, smoking, cardiovascular medications, exercise tolerance test metabolic equivalents
Logue	T2DM data drawn from a 2008 extract of the Scottish Care Information Diabetes Collaboration (>99% of people with diagnosed diabetes in Scotland)<br>*SCOTLAND*	BMI categories<br>20 to <25 kg/m^2<br>25 to <30 kg/m^2<br>30 to <35 kg/m^2<br>35 to <40 kg/m^2<br>40 to <45 kg/m^2<br>45 to <50 kg/m^2	Measurement of BMI recorded within one year of diagnosis-extracted from database	All-cause mortality	Data linked to mortality records (National Records of Scotland)	Age, smoking status
Ma	Korean Multicentre Cohort and participants from urban and rural areas in Korea<br>*SOUTH KOREA*	BMI categories<br><21 kg/m^2<br>21–22·9 kg/m^2<br>23–24·9 kg/m^2<br>≥25 kg/m^2	Anthropometric indices measured directly using standard methods during the baseline physical examination. Weight and height were measured to the nearest 0·5 kg and 0·5 cm	CVD mortality	Cause of death classified according to ICD-10. The national death certificate database used to identify deaths.	Age, blood pressure, alcohol consumption, smoking
McEwen	All participants from Translating Research Into Action for Diabetes (TRIAD) study with complete survey and medical record data<br>*USA*	BMI categories<br><26 kg/m^2<br>≥26 to <30 kg/m^2<br>≥30 to <35 kg/m^2<br>≥35 kg/m^2	BMI information extracted from patient surveys	All-cause mortality<br>CVD mortality	Deaths identified from National Death Index and verified by matching name, sex, date of birth and social security number	Unadjusted for sex-specific analysis (Odds Ratios estimated)<br>Age, sex, ethnicity, education, income, duration of diabetes, treatment of diabetes, smoking, hypertension, dyslipidaemia, macrovascular disease, retinopathy, nephropathy, peripheral neuropathy, Charlson index for the overall analysis
Menke	Patients with diabetes in NHANES III and 1999-2004 NHANES study. Participants with history of CVD or cancer, likely diagnosis of T1DM and who died during the first 2 years of follow-up excluded<br>*USA*	BMI categories<br>18·5–24·9 kg/m^2<br>25–29·9 kg/m^2<br>30–34·9 kg/m^2<br>≥35 kg/m^2	Weight measured using a digital scale to the nearest 0·01 kg and height measured using a stadiometer to the nearest 0·1 cm	All-cause mortality<br>CVD mortality	Study participants passively followed for mortality via linkage to the National Death Index. Cause of death was determined using the codes from ICD-9 and ICD-10.	Age, sex, ethnicity, smoking status, education, income, diabetes duration
Murphy	Participants with T2DM from the AGES-Reykjavik (a single-centre population-based cohort)<br>*ICELAND*	BMI categories<br>18·5–24·9 kg/m^2<br>25–29·9 kg/m^2<br>≥30 kg/m^2	Height and weight were determined using standardized protocols	All-cause mortality	Mortality ascertained from the Icelandic National Roster, an adjudicated registry of deaths.	Age, sex, education, duration of diabetes, midlife BMI, waist circumference, total and HDL cholesterol, systolic blood pressure, smoking status, hypertension, statin use, diabetes medication, CRP, microalbuminuria

ESM Table S1 (cont'd): Summary of the studies included in the meta-analysis

Study<br>First Author	Population<br>Sample	Exposure<br>definition	Exposure<br>assessment	Endpoint<br>definition	Endpoint<br>ascertainment	Confounders
Perotto	1565 persons with known T2DM resident in the town of Casale Monferrato (Casale Monferrato Study) with no cancer at baseline<br>*ITALY*	BMI quartiles<br><24·2 kg/m^2<br>24·3 to 26·7 kg/m^2<br>26·8 to 30 kg/m^2<br>>30 kg/m^2	Weight and height measured with participants not wearing shoes during diabetes clinic visits	All-cause mortality<br>CVD mortality	Deaths identified from registries of the town of residence, hospital discharge, and autopsy records. Cause defined with ICD-9 codes	Age, sex, smoking, diabetes duration, history of hypertension and CHD, albumin excretion rate, HbA1c, apoB/apoA1
Sluik	European Prospective Investigation into Cancer and Nutrition (EPIC; Cohort study from 10 European countries)<br>*MULTINATIONAL - EUROPE*	BMI quintiles<br>Men<br>≤24·9 kg/m^2<br>25·0–27·1 kg/m^2<br>27·2–29·1 kg/m^2<br>29·2–31·8 kg/m^2<br>≥31·9 kg/m^2<br>Women<br>≤24·7 kg/m^2<br>24·8–27·6 kg/m^2<br>27·7–30·3 kg/m^2<br>30·4–33·5 kg/m^2<br>≥33·6 kg/m^2	Weight and height measured with participants not wearing shoes. Each participant's body weight corrected in order to reduce heterogeneity due to different protocols in each site	All-cause mortality	Deaths identified with follow-up mailings and subsequent inquiries to municipality registries, regional health departments, physicians or hospitals.	Centre (stratified), age, diabetes duration, insulin treatment, self-reported history of MI, stroke, or cancer, smoking, educational level, physical activity, alcohol intake, quantiles of waist/height ratio
Thomas	Data from the General Practice Research Database (GPRD). Individuals with new diagnosis of T2DM between January 1990 and April 2007 (no prior CVD)<br>*UK*	BMI categories<br><25 kg/m^2<br>≥25 to <30 kg/m^2<br>>30 kg/m^2	Extracted from GPRD database	All-cause mortality	Death ascertained from modified GPRD death codes	Age, sex, smoking status, systolic and diastolic blood pressure, HbA1c, LDL and HDL cholesterol, triglycerides
Tobias	Population sample from two prospective cohort studies (Women: Nurse's Health Study; Men: Health Professionals Follow-up Study)<br>*USA*	BMI categories<br>18·5–22·4 kg/m^2<br>22·5–24·9 kg/m^2<br>25·0–27·4 kg/m^2<br>27·5–29·9 kg/m^2<br>30·0–34·9 kg/m^2<br>≥35·0 kg/m^2	Self-reported weight and height from questionnaires [sensitivity analysis to assess the potential effect of error in self-reporting]	All-cause mortality	Reports by the next of kin or postal authorities or from searches of the National Death Index.	Age, race, marital status, menopausal status, family history of diabetes, smoking status, alcohol intake, healthy eating index score, physical activity
Tseng	Population from the compulsory and universal National Health Insurance in Taiwan since March 1995<br>*TAIWAN*	BMI categories<br><18·5 kg/m^2<br>18·5–22·9 kg/m^2<br>23·0–24·9 kg/m^2<br>25·0–29·9 kg/m^2<br>≥30 kg/m^2	Self-reported BMI extracted from questionnaire	All-cause mortality	Registries and death certificates	Age, diabetes duration, insulin use, hypertension, smoking, living region, sex (for the unstratified analysis)
Tuomilehto	Random sample of 1100 households from electoral list by the National Department of Statistics. Survey performed in subjects >15 years old in a sample of 3040<br>*MALTA*	BMI quartiles<br>Men<br>17·1–24·5 kg/m^2<br>24·6–26·9 kg/m^2<br>27·0–29·6 kg/m^2<br>29·7–47·3 kg/m^2<br>Women<br>12·5–26·0 kg/m^2<br>26·1–29·4 kg/m^2<br>29·5–33·0 kg/m^2<br>33·1–72·1 kg/m^2	Weight and height measured on daily calibrated scales, with the subject lightly dressed and without shoes	All-cause mortality	Deceased cases were registered during the follow-up.	Unadjusted (Odds Ratios estimated)
Zhao	Cohort of diabetes patients (Louisiana State University Hospital based Longitudinal Study database)<br>*USA*	BMI categories<br>18·5–22·9 kg/m^2<br>23–24·9 kg/m^2<br>25–29·9 kg/m^2<br>30–34·9 kg/m^2<br>35–39·9 kg/m^2<br>≥40 kg/m^2	Data for BMI extracted from computerized hospitalisation records. Height was measured without shoes and weight with light clothing	All-cause mortality	Mortality outcomes assessed by linkage with the Stage Centre for Health Statistics at the Louisiana Office of Public Health.	Age, sex, smoking, income, type of insurance, HbA1C, LDL cholesterol, systolic blood pressure, eGFR, use of antihypertensive, glucose-lowering and cholesterol-lowering agents
Zoppini	Population from Verona Diabetes Study. BMI available in medical notes<br>*ITALY*	BMI quartiles<br>Patients <65 years<br>≤ 25·4 kg/m^2<br>25·5-27·9 kg/m^2<br>28·0-30·8 kg/m^2<br>≥30·9 kg/m^2<br>Patients ≥65 years<br>≤ 24·6 kg/m^2<br>24·7–26·9 kg/m^2<br>27·0–29·8 kg/m^2<br>≥29·9 kg/m^2	BMI information extracted from medical records	All-cause mortality<br>CVD mortality	Data obtained from death certificates using death codes.<br>Causes of death grouped according to ICD-9.	Age, sex, smoking, fasting plasma glucose, hypertension, diabetes duration and treatment

ESM Table S2: Prevalence of cardiovascular disease at baseline, diabetes definition, and exclusion of first years of follow-up in the analysis

Study<br>First Author	Baseline<br>CVD	Diabetes<br>Type	First years<br>excluded	Diabetes<br>Definition
Bozorgmanesh	Yes	NR	NR	Self-reported physician-diagnosed diabetes; use of glucose lowering agents; ADA criteria (2003)
Chaturvedi	No	2	NR	Excluded: subjects requiring insulin treatment within 1 year
Church	No	1 & 2	NR	Physician-diagnosed diabetes, insulin therapy, FPG ≥7mmol/l (≥126mg/dl)
Costanzo	No	2	2 years[†]	Physician-diagnosed diabetes
Eeg-Olofsson	No	2	NR	Treatment with: diet or oral glucose-lowering drugs only; insulin ± oral glucose-lowering drugs only and ≥40 years at diagnosis
Jackson	No	2	NR	Excluded: <25 years and on insulin treatment
Khalangot	No	2	6 months[†]	WHO criteria (1985); EASD/IDF criteria (1999); Insulin ± oral glucose-lowering drugs
Kokkinos	Yes	2	NR	ICD-codes
Logue	NR	2	2 years[^]	Excluded: <30 years at diagnosis
Ma	NR	NR	NR	Hospital diagnosis, drug treatment, FPG ≥7mmol/l (≥126mg/dl)
McEwen	Yes	NR	NR	Surveys, medical record reviews, and administrative data
Menke	No	2	2 years[^]	Self-reported, ADA criteria (2010). Excluded: <30 years at diagnosis *and* current insulin use *and* insulin therapy 1 year after diagnosis
Murphy	Yes	2	2 years[*]	Self-reported, glucose-lowering drugs, FPG ≥7mmol/l (≥126mg/dl)
Perotto	Yes	2	2 years[†]	Physician-diagnosed diabetes, hospital discharge records, registries of drugs, syringes, and reagent strips prescriptions
Sluik	Yes	NR	NR	Self-reported; contact with general practitioner; self-reported glucose-lowering drugs; linkage to diabetes registries; HbA1c>6%
Thomas	No	2	2 years[^]	Validated computerized algorithm applied to the General Practice Research Database
Tobias	No	2	4 years[**]	Mail-confirmed physician diagnosis; Diabetes Data Group Classification (1998)
Tseng	NR	2	2 years[*]	National Health Insurance Database
Tuomilehto	NR	NR	NR	WHO criteria (1980 and 1985)
Zhao	No	2	2 years[**]	ICD-codes (physician-validated using ADA criteria (1997))
Zoppini	NR	2	4 years[†]	Patients identified from general practitioner, diabetes physicians, and drug consumption database (population-based survey)

ADA: American Diabetes Association
CVD: Cardiovascular disease
NR: Not reported

† Sensitivity analyses with the same results, data not reported
^ Primary analysis
* Sensitivity analyses with complete data reported (used in the analysis)
** Sensitivity analyses without complete data reported

ESM Table S3: Newcastle-Ottawa Quality Scores for the studies included in the meta-analysis

	Newcastle-Ottawa Scores			
Study First Author	Selection (1-4)	Comparability (1-2)	Outcome (1-3)	Total Score (0-9)
Bozorgmanesh	4	1	2	7
Chaturvedi	4	2	3	9
Church	3	0	2	5
Costanzo	4	2	2	8
Eeg-Olofsson	4	2	2	8
Jackson	2	2	2	6
Khalangot	4	2	1	7
Kokkinos	4	2	2	8
Logue	4	2	1	7
Ma	4	2	2	8
McEwen	3	2	1	6
Menke	4	2	3	9
Murphy	4	2	2	8
Perotto	3	2	3	8
Sluik	4	2	2	8
Thomas	4	2	2	8
Tobias	2	2	2	6
Tseng	3	2	2	7
Tuomilehto	4	0	1	5
Zhao	4	2	2	8
Zoppini	4	2	2	8

The Newcastle-Ottawa Scale (NOS) for assessing the quality of nonrandomised studies in meta-analyses. Available from http://www.ohri.ca/programs/clinical_epidemiology/oxford.asp

ESM Table S4: Heterogeneity of associations between BMI and outcomes

Outcome	Sex	No. of Studies	I^2 *(95% CI)*	
			**BMI-1**	**BMI-2**
All-cause mortality	Both	18	92 (74, 96)	62 (20, 81)
	Men	9	96 (60, 99)	85 (11, 95)
	Women	8	91 (35, 97)	70 (4, 89)
Cardiovascular mortality	Both	6	2 (0, 48)	45 (0, 78)

BMI-1 and BMI-2 indicate the two spline transformations of BMI

ESM Table S5: Exclusion of studies reporting unadjusted estimates

Sex	Outcome	Excluded McEwen	Excluded Tuomilehto	Test for non-linearity	Nadir BMI (kg/m^2)
Both sexes	All-cause mortality	No	Yes	<0.001	33.8
Men	All-cause mortality	Yes	Yes	0.007	34.8
Women	All-cause mortality	Yes	Yes	<0.001	31.5

As shown in Table 1 and Table S1, McEwen reported adjusted analysis for combined men and women and unadjusted analysis stratified by sex, for both outcomes; Tuomilehto reported unadjusted analyses stratified by sex but only for all-cause mortality.

ESM Table S6: Subgroup analyses

Stratification	Study	P for heterogeneity
**By geographical region**		
US	Jackson, McEwen, Menke, Tobias, Zhao, Chaturvedi (1 cohort)	0.069
Europe and UK	Eeg-Olofsson, Khalangot, Murphy, Perotto, Sluik, Tuomilehto, Zoppini, Costanzo, Logue, Thomas, Chaturvedi (1 cohort)	
Asia	Bozorgmanesh, Tseng, Chaturvedi (1 cohort)	
**By study period**		
2003-2009	Eeg-Olofsson, Khalangot, McEwen, Zoppini	0.117
2011-2013	Logue, Perotto, Sluik, Tseng	
2014-2015	Bozorgmanesh, Costanzo, Jackson, Menke, Murphy, Thomas, Tobias, Zhao	

We investigated heterogeneity only for the association between BMI with all-cause death in both sexes, given the few studies with data stratified by sex and on cardiovascular mortality (Table 1). For the “study period” subgroup analysis, we excluded two studies before 2000: Tuomilehto (1994) and Chaturvedi (1995)

ESM Figure S1: Study-specific estimates for all-cause mortality, both sexes

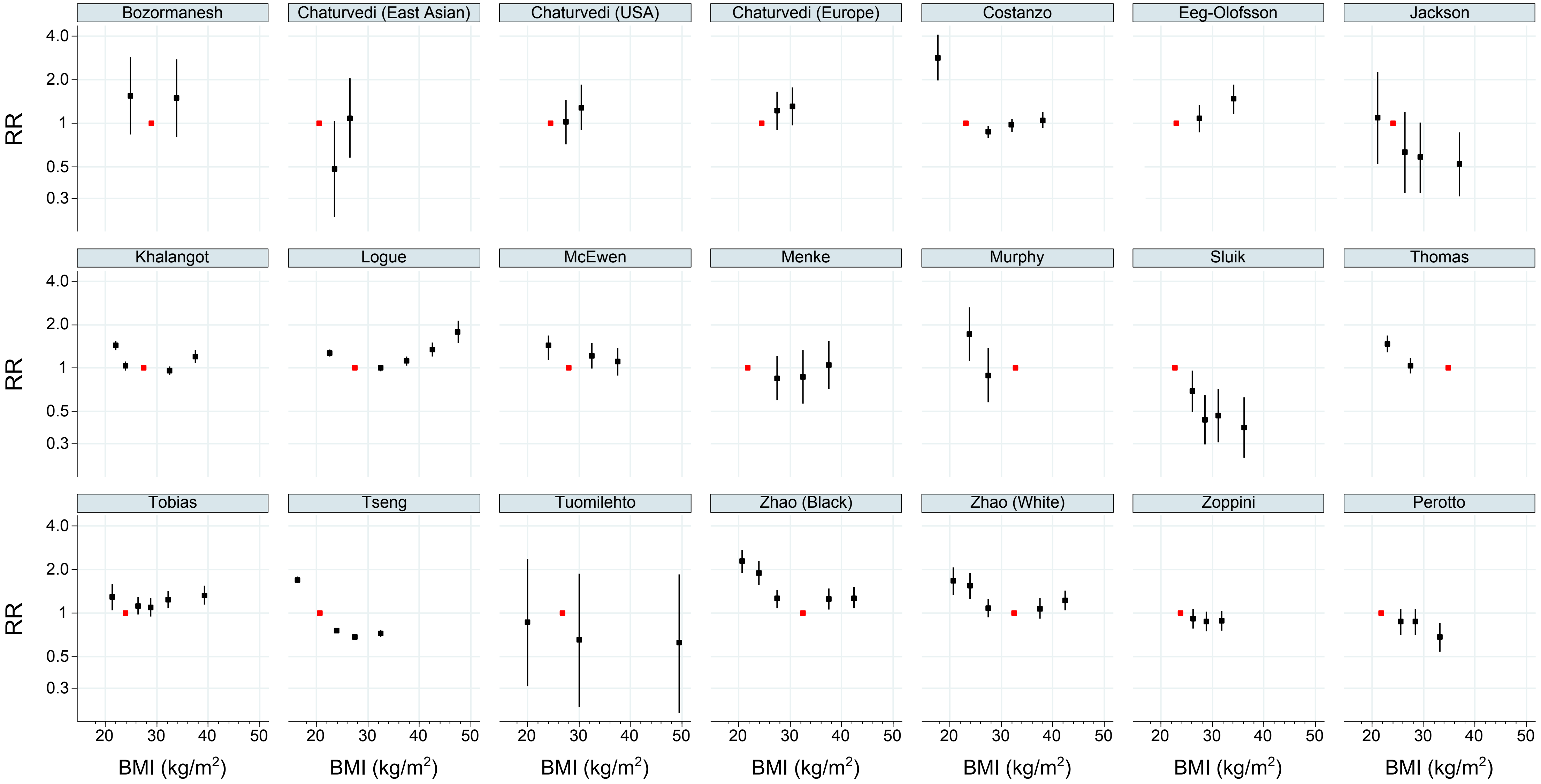

Red squares indicate study-specific references (RR 1) and bars 95% CIs. RR: Relative Risk.

ESM Figure S2: Study-specific estimates for all-cause mortality, men

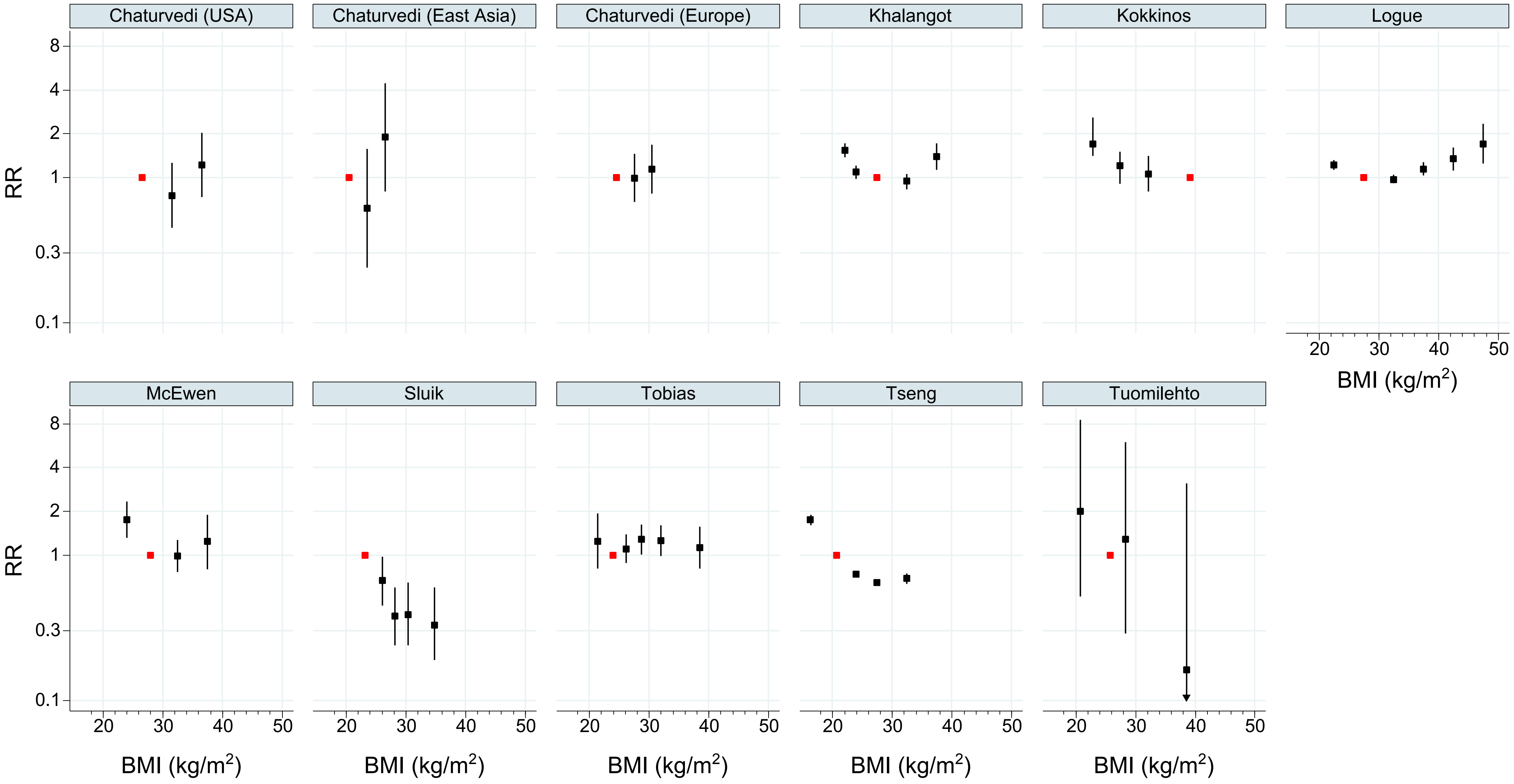

Red squares indicate study-specific references (RR 1) and bars 95% CIs. RR: Relative Risk.

ESM Figure S3: Study-specific estimates for all-cause mortality, women

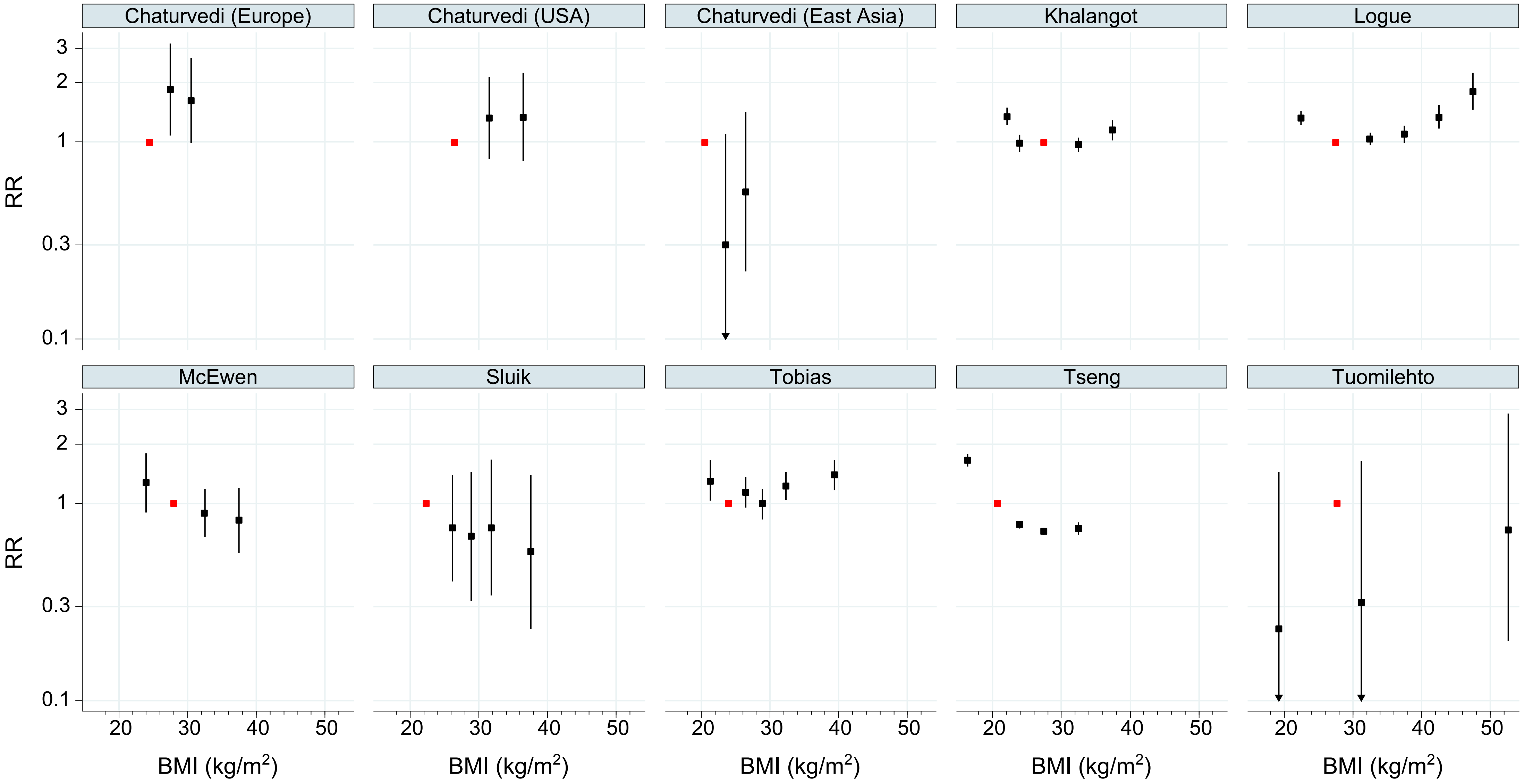

Red squares indicate study-specific references (RR 1) and bars 95% CIs. RR: Relative Risk

ESM Figure S4: Study-specific estimates for cardiovascular mortality, both sexes

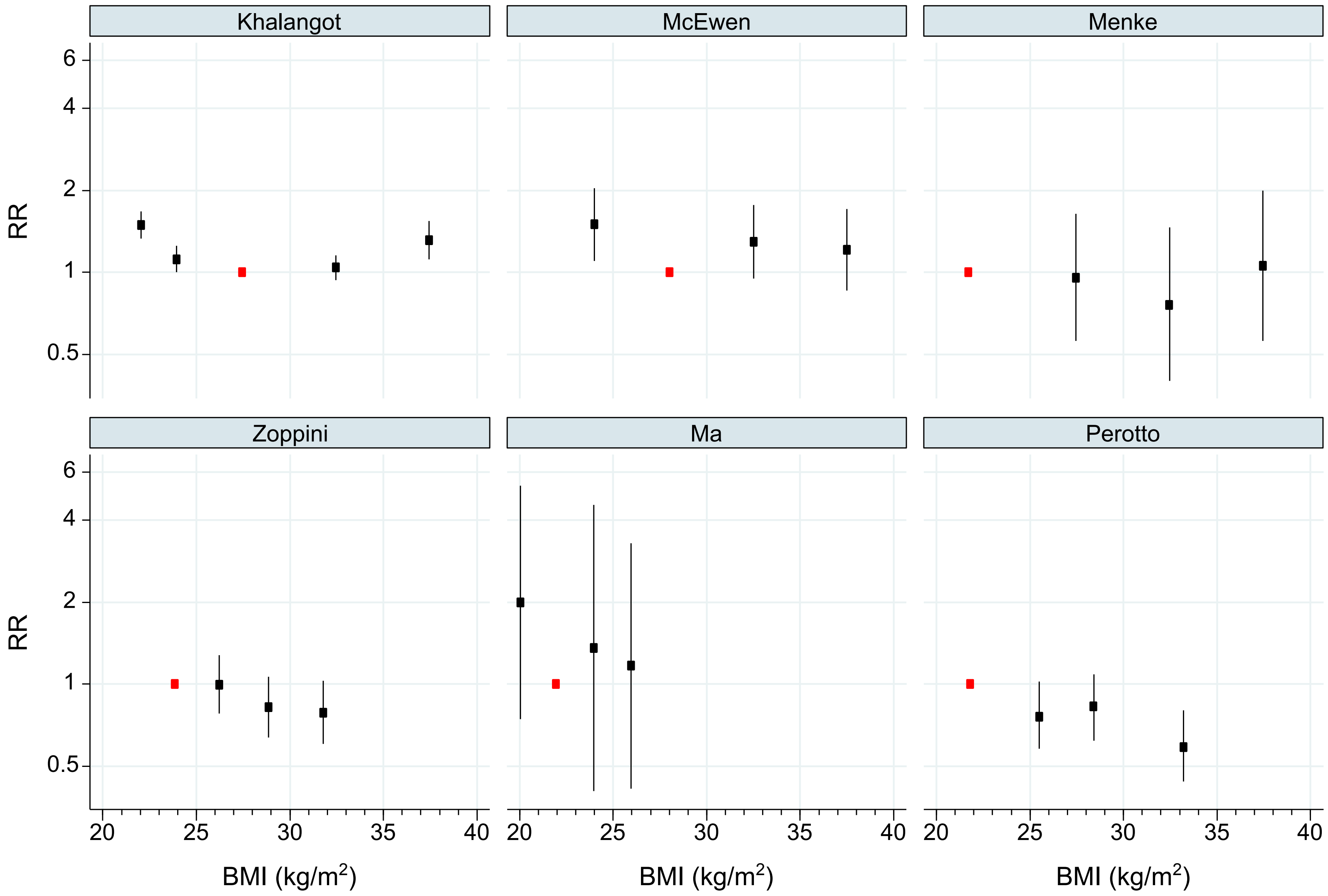

Red squares indicate study-specific references (RR 1) and bars 95% CIs. RR: Relative Risk.

ESM Figure S5: Relationship between BMI and all-cause mortality excluding studies with unadjusted estimates

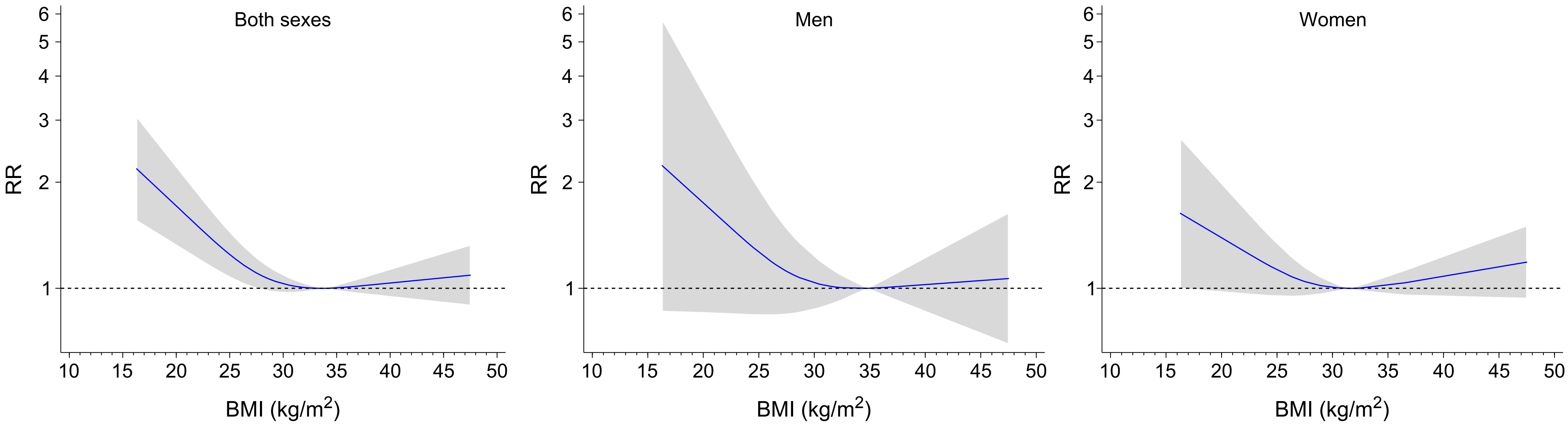

As for cardiovascular mortality it was not possible to stratify the analysis by sex (few studies), sensitivity analyses could be done only for combined men and women. However, McEwen reported adjusted analysis for combined sexes, while Tuomilehto reported no data for cardiovascular mortality. Therefore, no sensitivity analyses could be performed for cardiovascular mortality.
Grey areas indicate 95% CIs. RR: Relative Risk.

ESM Figure S6: Relationship between BMI and all-cause mortality in both sexes, by geographical region

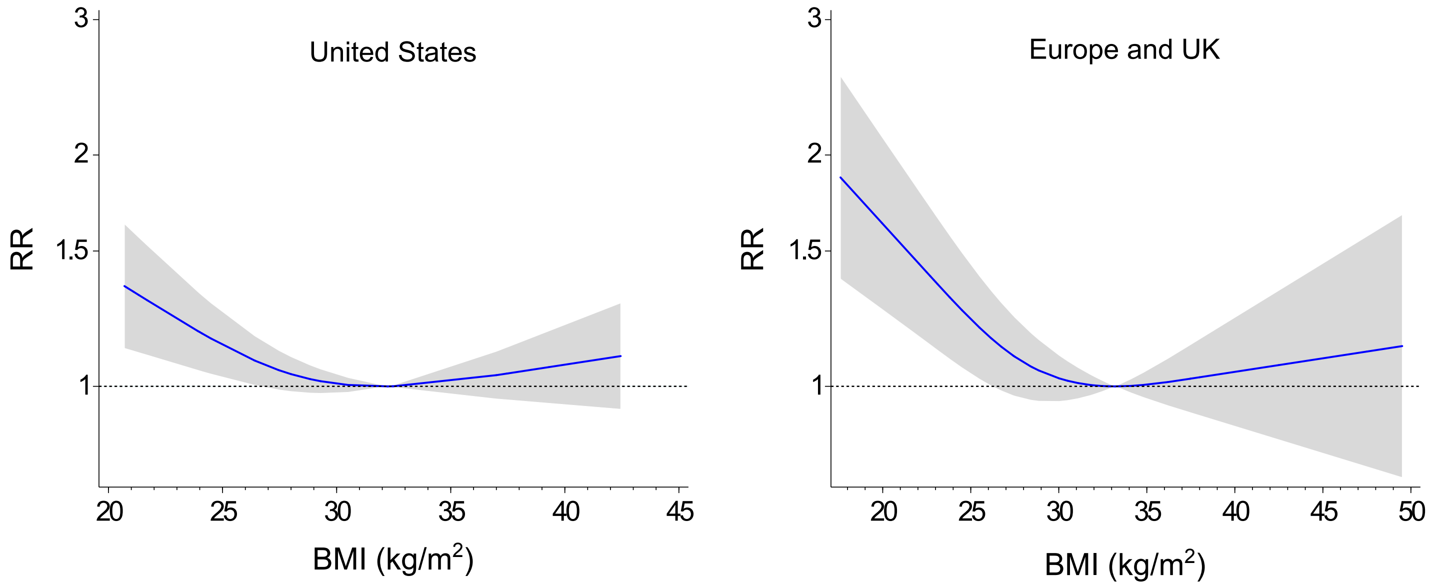

Data not shown for Asian populations given the few studies available (n=3). Grey areas indicate 95% CIs. RR: Relative Risk.

ESM Figure S7: Relationship of BMI with all-cause and cardiovascular mortality using knots at 25th, 50th, and 75th percentile of BMI distribution

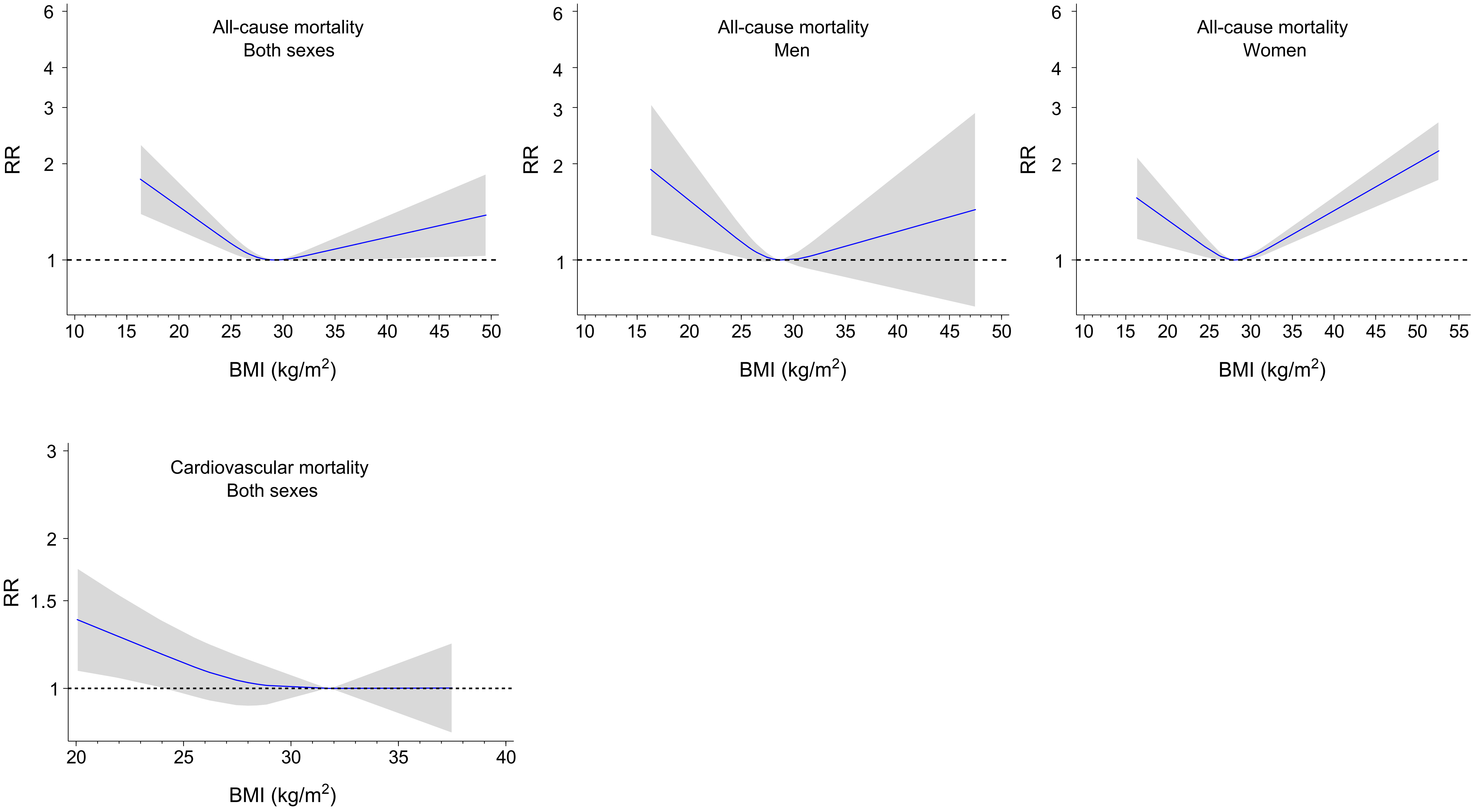

Grey areas indicate 95% CIs. RR: Relative Risk.

ESM Figure S8: Relationship of BMI with all-cause mortality (both sexes) using different knot positions

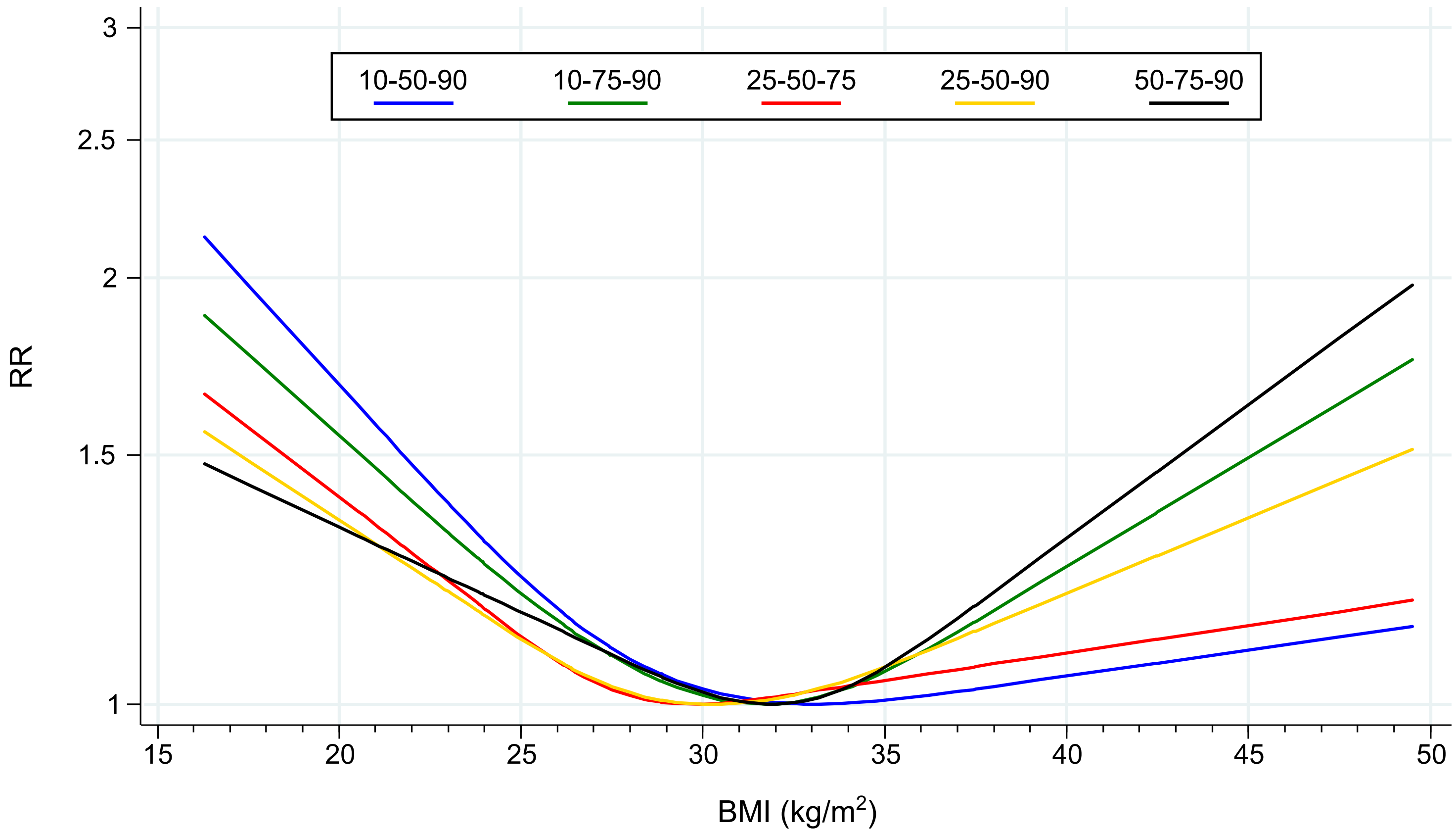

Different colours indicate knots percentile positions of BMI distribution; RR: Relative Risk.
